# Supplementary material for: Dissociation rate compensation mechanism for budding yeast pioneer transcription factors
Source: eLife. 2019 Mar 19;8:e43008. doi: 10.7554/eLife.43008 (PMC6449090; doi:10.7554/eLife.43008)
Supplement: Supplementary file 1. [file elife-43008-supp1.docx]

**Supplemental Tables**

**Table S1: All binding affinity measurements**

| TF | Substrate | Experiment | S_1/2_ (nM) | # replicates |
| --- | --- | --- | --- | --- |
| Reb1 | 25 bp DNA | EMSA | 2.3 ± 0.2 | 2 |
| Reb1 | 25 bp DNA (- site) | EMSA | 21.7 ± 2.3 | 2 |
| Reb1 | 94 bp DNA | Ensemble PIFE | 5.1 ± 0.2 | 3 |
| Reb1 | DNA | smPIFE primary | 18.1 ± 3.0 | 3 |
| Reb1 | DNA | smPIFE secondary | 1.2 ± 0.2 | 3 |
| Reb1 | Nuc (P3) | EMSA | 4.6 ± 0.1 | 2 |
| Reb1 | Nuc (P8) | EMSA | 1.5 ± 0.1 | 2 |
| Reb1 | Nuc (P13) | EMSA | 8.5 ± 0.2 nM | 2 |
| Reb1 | Nuc (P18) | EMSA | 11.2 ± 0.3 nM | 2 |
| Reb1 | Nuc (P-) | EMSA | 32.7 ± 0.8 | 2 |
| Reb1 | Nuc (P3) | FRET | 7.9 ± 1.3 | 3 |
| Reb1 | Nuc (P8) | FRET | 2.4 ± 0.3 | 3 |
| Reb1 | Nuc (P13) | FRET | 101.5 ± 19.1 | 3 |
| Reb1 | Nuc (P18) | FRET | NA | 3 |
| Reb1 | Nuc (P-) | FRET | NA | 3 |
| Reb1 | Nuc (P8) | smFRET primary | 7.3 ± 1.5 | 3 |
| Reb1 | Nuc (P8) | smFRET secondary | 116 ± 39 | 3 |
| Reb1 ΔN | 94 bp DNA | PIFE | 7.8 ± 0.5 | 3 |
| Reb1 ΔN | Nuc (P8) | FRET | 11.8 ± 0.9 | 3 |
| Cbf1 | DNA | EMSA | 1.07 ± 0.01 | 2 |
| Cbf1 | DNA | PIFE | 1.3 ± 0.3 | 3 |
| Cbf1 | DNA | smPIFE primary | 12.3 ± 3.5 | 3 |
| Cbf1 | DNA | smPIFE secondary | 1.4 ± 0.4 | 3 |
| Cbf1 | Nuc | EMSA | 11.5 | 1 |
| Cbf1 | Nuc (P8) | FRET | 12.3 ± 1.6 | 3 |
| Cbf1 | Nuc (P8) | smFRET | 52.9 ± 6.0 | 3 |

**Table S2: rates from single molecule experiments**

| TF | Substrate | k_on primary_ (s^-1^ nM^-1^) | k_off primary_ (s^-1^) | k_on secondary_ (s^-1^) | k_off secondary_ (s^-1^) |
| --- | --- | --- | --- | --- | --- |
| Reb1 | DNA | 0.032 ± 0.003 | 0.58 ± 0.08 | 0.022 ± 0.002 | 0.036 ± 0.005 |
| Reb1 | Nuc | 0.0006 ± 0.0001 | 0.0044 ± 0.0005 | NA | 0.07 ± 0.02 |
| Reb1 ΔN | Nuc | 0.0003 ± 0.00001 | 0.0044 ± 0.0008 | NA | 0.15 ± 0.08 |
| Cbf1 | DNA | 0.025 ± 0.006 | 0.3 ± 0.05 | 0.024 ± 0.003 | 0.034 ± 0.004 |
| Cbf1 | Nuc | 0.00021 ± 0.00002 | 0.0111 ± 0.0007 | NA | NA |

**Table S3:**

**table of relative binding affinities (S_1/2 Nuc_/S_1/2 DNA_)**

| Realative binding affinity : single molecule vs ensemble | | |
| --- | --- | --- |
|  | single molecule | Ensemble |
| Reb1 Primary | 0.40 ± 0.03 | 0.47 ± 0.01 |
| Cbf1 Primary | 4.3 ± 1.3 | 9.5 ± 2.5 |

**Table S4: Primers used for *in vitro* experiments**

| **Reb1 Primers** | Binding motif: CCGGGTAAC |
| --- | --- |
| P- Fwd | Cy3-CTGGAGAATCCCGGTGCCGAGGCCGC |
| P3 Fwd | Cy3-CTCCGGGTAACCGGTGCCGAGGCCGC |
| P8 Fwd | Cy3-CTGGAGACCGGGTAACCCGAGGCCGC |
| P13 Fwd | Cy3-CTGGAGAATCCCCCGGGTAACGCCGC |
| P18 Fwd | Cy3-CTGGAGAATCCCGGTGCCCGGGTAACTCAATTGGTCG |
| Slide | GACACTGGGACATGCATCGG[\Cy3]TGGAGAATCCCGG |
| Slide Rvs | ACAGGATGTTACCCGGTGACACGTGCCTGGAGACTAGGG |
| 25 bp +site | Cy3-TCCGGGTAACCGGTGCCGAGGCCGC |
| 25 bp - site | Cy3-TGGAGAATCCCGGTGCCGAGGCCGC |
| smPIFE Fwd | Cy3-ACCGGGTAACCGGTGCCGAGGCCGC |
| smPIFE Rvs | [BTN]-CGCCTAACACGCGGGGG[\Cy5]CAGCGCG |
| 147 Nuc Rvs | ACAGGATGTATATATCTGACACGTGCCTGG |
| 222 Rvs | [BTN]-CGCATGCTGCAGACGCGTT |
| **Cbf1 Primers** | Binding motif: GGTCACGTGACC |
| Cbf1 P8 | Cy3-CTGGAGAGGTCACGTGACCAGGCCGCTC |
| Cbf1 smPIFE Rvs | [BTN]-GCGGTTAAAAC[\Cy5]CGGGGGACA |
| Cbf1 smPIFE Fwd | Cy3-GGTCACGTGACCTGCCGAGGCCGCTC |

**Table S5: single molecule quality control data**

| TF | Substrate | [TF] (nM) | Acquisition rate (Hz) | Number of replicates | % fluctuating |
| --- | --- | --- | --- | --- | --- |
| Reb1 | DNA | 2 | 5 | 3 | 10 |
| Reb1 | DNA | 5 | 5 | 3 | 13 |
| Reb1 | DNA | 10 | 5 | 3 | 15 |
| Reb1 | DNA | 15 | 5 | 2 | 7 |
| Reb1 | Nuc (P8) | 2 | 0.5 | 3 | 30 |
| Reb1 | Nuc (P8) | 5 | 0.5 | 3 | 44 |
| Reb1 | Nuc (P8) | 10 | 0.5 | 3 | 63 |
| Reb1 | Nuc (P8) | 15 | 0.5 | 3 | 38 |
| Reb1 ΔN | Nuc (P8) | 5 | 0.5 | 3 | 27 |
| Reb1 ΔN | Nuc (P8) | 10 | 0.5 | 3 | 44 |
| Reb1 ΔN | Nuc (P8) | 20 | 0.5 | 3 | 47 |
| Reb1 ΔN | Nuc (P8) | 30 | 0.5 | 3 | 55 |
| Cbf1 | DNA | 1 | 5 | 3 | 12 |
| Cbf1 | DNA | 2 | 5 | 3 | 15 |
| Cbf1 | DNA | 5 | 5 | 3 | 17 |
| Cbf1 | DNA | 10 | 5 | 3 | 13 |
| Cbf1 | Nuc (P8) | 10 | 0.5 | 3 | 35 |
| Cbf1 | Nuc (P8) | 20 | 0.5 | 3 | 30 |
| Cbf1 | Nuc (P8) | 30 | 0.5 | 5 | 31 |
| Cbf1 | Nuc (P8) | 40 | 0.5 | 5 | 26 |

**Table S6: Primers used for *in vivo* experiments**

| **primers** | **Sequence** | **Description** |
| --- | --- | --- |
| PF1-STH1_GFP | AAATGAGTTTACTGATGAATGGTTCAAGGAACACTCTTCGaGTAAAGGAGAAGAACTTTT | primer used to amplify GFP-HIS3MX cassette |
| PR1-STH1_TEFt | TAGTCGTAAAAAAAAAAAACATGTGGTGATGAAAACGTTAcAGTATAGCGACCAGCATTC | primer used to amplify GFP-HIS3MX cassette |
| PF1-HHT2_GFP | GGATATCAAATTGGCCAGAAGACTAAGAGGTGAAAGATCAAGTAAAGGAGAAGAACTTTT | primer used to amplify GFP-HIS3MX cassette |
| PR1-HHT2_TEFt | CTAAATGCATAGAAAAAAAAAAATTCCCGCTTTATATTCACAGTATAGCGACCAGCATTC | primer used to amplify GFP-HIS3MX cassette |
| PF1-REB1(YDY 11) | GTATGGGCGGTACGAGATC | primer used to amplify GFP-HIS3MX cassette |
| PR1-REB1(YDY 14) | GACTGATGAGTTCCACCTGC | primer used to amplify GFP-HIS3MX cassette |
| PF1-NHP6A_GFP | ATATGAATCCGAAAAGGAGTTATATAACGCCACTTTGGCTAGTAAAGGAGAAGAACTTTT | primer used to amplify GFP-HIS3MX cassette |
| PR1-NHP6A_TEFt | TAAATCACACAGACAAAAACGCGGGGAGGAAGTATCCCTACAGTATAGCGACCAGCATTC | primer used to amplify GFP-HIS3MX cassette |
| PF1-NHP6A | GGGTGAGAAGTGGAAGGCTC | primer used to check the integration of the GFP-HIS3MX cassette |
| PF1-HHT2 | ATCTGGCTGCTATTCACGCT | primer used to check the integration of the GFP-HIS3MX cassette |
| PF2-STH1 | AGCCAGAGAGTAAATCGCCC | primer used to check the integration of the GFP-HIS3MX cassette |
| PR-AdHt | GCTTATTTAGAAGTGGCGCG | primer used to check the integration of the GFP-HIS3MX cassette |
