# Supplementary material for: Dissociation rate compensation mechanism for budding yeast pioneer transcription factors
Source: eLife. 2019 Mar 19;8:e43008. doi: 10.7554/eLife.43008 (PMC6449090; doi:10.7554/eLife.43008)
Supplement: Transparent reporting form [file elife-43008-transrepform.docx]

***eLife’s* transparent reporting form**

We encourage authors to provide detailed information *within their submission* to facilitate the interpretation and replication of experiments. Authors can upload supporting documentation to indicate the use of appropriate reporting guidelines for health-related research (see [EQUATOR Network](http://www.equator-network.org/%20)), life science research (see the [BioSharing Information Resource](https://biosharing.org/)), or the [ARRIVE guidelines](http://www.plosbiology.org/article/info:doi/10.1371/journal.pbio.1000412) for reporting work involving animal research. Where applicable, authors should refer to any relevant reporting standards documents in this form.

If you have any questions, please consult our Journal Policies and/or contact us: [editorial@elifesciences.org](mailto:editorial@elifesciences.org).

**Sample-size estimation**

- You should state whether an appropriate sample size was computed when the study was being designed
- You should state the statistical method of sample size computation and any required assumptions
- If no explicit power analysis was used, you should describe how you decided what sample (replicate) size (number) to use

Please outline where this information can be found within the submission (e.g., sections or figure legends), or explain why this information doesn’t apply to your submission:

For single molecule measurements, the sample size was not predetermined. We subjected all visible molecules to the selection criteria described in the methods. Additionally, we confirmed that the molecules analyzed were representative of the entire population by comparing our results to ensemble measurements (Reb1: figure 4- figure supplement 1E, Cbf1: figure 5-figure supplement 1G) .

**Replicates**

- You should report how often each experiment was performed
- You should include a definition of biological versus technical replication
- The data obtained should be provided and sufficient information should be provided to indicate the number of independent biological and/or technical replicates
- If you encountered any outliers, you should describe how these were handled
- Criteria for exclusion/inclusion of data should be clearly stated
- High-throughput sequence data should be uploaded before submission, with a private link for reviewers provided (these are available from both GEO and ArrayExpress)

Please outline where this information can be found within the submission (e.g., sections or figure legends), or explain why this information doesn’t apply to your submission:

For single molecule measurements: the number of analyzed molecules in this study is included in the supplement and is consistent with previous studies (see PMC3950707). The criteria for exclusion is explained in the methods section. Additionally, the supplemental text file reports the percentage of traces with colocalized cy3/cy5 signal analyzed for smFRET and smPIFE experiments. The number of replicates reported are technical replicates. For each experiment, a single biological replicate was performed. For ensemble measurements: the number of independent technical replicates is reported in the supplement.

**Statistical reporting**

- Statistical analysis methods should be described and justified
- Raw data should be presented in figures whenever informative to do so (typically when N per group is less than 10)
- For each experiment, you should identify the statistical tests used, exact values of N, definitions of center, methods of multiple test correction, and dispersion and precision measures (e.g., mean, median, SD, SEM, confidence intervals; and, for the major substantive results, a measure of effect size (e.g., Pearson's r, Cohen's d)
- Report exact p-values wherever possible alongside the summary statistics and 95% confidence intervals. These should be reported for all key questions and not only when the p-value is less than 0.05.

Please outline where this information can be found within the submission (e.g., sections or figure legends), or explain why this information doesn’t apply to your submission:

For single-molecule measurements, kinetic rates were determined by fitting dwell time distributions to exponential distributions. The proper fit was determined through a log-liklihood ratio test using MEMLET (PMC4968482). P-values and criteria for determining proper fit are documented in the supplement. In figure 6-figure supplement 1, where we compare isoelectric points of pioneer factors vs other TFs, p-values are reported in the figure and were calculated using a Student's t test using JMP (www.jmp.com). For all measurements error bars represent one standard error of the mean.

(For large datasets, or papers with a very large number of statistical tests, you may upload a single table file with tests, Ns, etc., with reference to sections in the manuscript.)

**Group allocation**

- Indicate how samples were allocated into experimental groups (in the case of clinical studies, please specify allocation to treatment method); if randomization was used, please also state if restricted randomization was applied
- Indicate if masking was used during group allocation, data collection and/or data analysis

Please outline where this information can be found within the submission (e.g., sections or figure legends), or explain why this information doesn’t apply to your submission:

No group allocation was performed

**Additional data files (“source data”)**

- We encourage you to upload relevant additional data files, such as numerical data that are represented as a graph in a figure, or as a summary table
- Where provided, these should be in the most useful format, and they can be uploaded as “Source data” files linked to a main figure or table
- Include model definition files including the full list of parameters used
- Include code used for data analysis (e.g., R, MatLab)
- Avoid stating that data files are “available upon request”

Please indicate the figures or tables for which source data files have been provided:

Supplementary figures include all replicate gels used in these experiments. Tables of binding equilibria and rates from ensemble and single-molecule measurements are included in supplement.
